# Human Pluripotent Stem Cells in Reproductive Science—A Comparison of Protocols Used to Generate and Define Male Germ Cells from Pluripotent Stem Cells

**DOI:** 10.3390/ijms21031028

**Published:** 2020-02-04

**Authors:** Magdalena Kurek, Halima Albalushi, Outi Hovatta, Jan-Bernd Stukenborg

**Affiliations:** 1 NORDFERTIL Research Lab Stockholm, Childhood Cancer Research Unit, Department of Women’s and Children’s Health, Karolinska Institutet, and Karolinska University Hospital, 17164 Solna, Sweden; magdalena.kurek@ki.se (M.K.); halima07@squ.edu.om (H.A.); 2 College of Medicine and Health Sciences, Sultan Qaboos University, 123 Muscat, Oman; 3 Department of Clinical Science, Intervention and Technology (CLINTEC), Karolinska Institutet and University Hospital Karolinska Institutet, 141 52 Huddinge, Sweden; outi.hovatta@kolumbus.fi

**Keywords:** embryonic stem cells, induced pluripotent stem cells, pluripotent stem cells, male germ cells, differentiation, infertility

## Abstract

Globally, fertility-related issues affect around 15% of couples. In 20%–30% of cases men are solely responsible, and they contribute in around 50% of all cases. Hence, understanding of in vivo germ-cell specification and exploring different angles of fertility preservation and infertility intervention are considered hot topics nowadays, with special focus on the use of human pluripotent stem cells (hPSCs) as a source of in vitro germ-cell generation. However, the generation of male germ cells from hPSCs can currently be considered challenging, making a judgment on the real perspective of these innovative approaches difficult. Ever since the first spontaneous germ-cell differentiation studies, using human embryonic stem cells, various strategies, including specific co-cultures, gene over-expression, and addition of growth factors, have been applied for human germ-cell derivation. In line with the variety of differentiation methods, the outcomes have ranged from early and migratory primordial germ cells up to post-meiotic spermatids. This variety of culture approaches and cell lines makes comparisons between protocols difficult. Considering the diverse strategies and outcomes, we aim in this mini-review to summarize the literature regarding in vitro derivation of human male germ cells from hPSCs, while keeping a particular focus on the culture methods, growth factors, and cell lines used.

## 1. Introduction

Since the first derivation of human embryonic stem cells (hESCs) in 1998 [1], stem-cell approaches are among the most favourable systems to model in vivo developmental processes, as they allow differentiation into all three germ layers. Further, the breakthrough in the derivation of patient-specific human induced pluripotent stem cells (hiPSCs) by reprogramming a patient’s own somatic cells [2] into a human pluripotent stem cell (hPSC) state is pushing the involvement of hPSCs towards use in regenerative medicine. 

The latest generation of several disease-specific hiPSC lines, e.g., Klinefelter syndrome [3,4,5], Mowat–Wilson syndrome [6], Dravet syndrome [7], or patients with non-obstructive azoospermia (NOA) [8] might help to understand fundamental aspects of these diseases and might help to establish new treatment strategies.

The lack of treatment options for men suffering germ-cell loss-related infertility as well as the still inadequate level of knowledge of human embryonic germ-cell development, mainly limited as a result of inaccessibility of experimental material, results in a big interest in in vitro derivation of germ cells from hPSCs [9,10,11]. Differentiation of hPSCs towards germ cells in vitro was first observed as spontaneous differentiation into embryoid bodies (EBs), where the expression of DEAD-box helicase 4 (DDX4) indicated the presence of germ-cell-like cells [12]. Nevertheless, the reconstitution of human germ-cell development in vitro is challenging, as human in vivo development, with all its different cues and signals, is not yet fully understood. Therefore, most current in vitro differentiation approaches trying to mimic the in vivo situation mostly reflect knowledge extrapolated from rodent studies. In a study from 2018, Zhao and colleagues reported the differentiation of H1 hESCs, as well as of hiPSCs established from skin biopsy material of patients with NOA, into haploid germ cells [8]. The protocol used in this study was based on the protocol published by Easley and colleagues in 2012 [13]. By omitting 2-mercaptoethanol and bovine serum albumin, and with the addition of xeno-free serum replacement and vitamin C to the culture medium, the modified protocol demonstrated a reduction in the number of apoptotic cells and a differentiation efficiency of >1% of meiotic as well as >5% of haploid cells in the fraction of differentiated cells could be observed [14]. In addition, to the supportive effect of vitamin C and the omission of 2-mercaptoethanol, the study highlighted the importance of nanos C2HC-type zinc finger 3 (NANOS3) in relation to the differentiation into spermatogonial stem cell-like cells.

As extrapolated from murine studies, specification of primordial germ cells (PGCs) from the epiblast in response to bone morphogenetic protein (BMP) signalling from extraembryonic tissue is the first step in human germ-cell development [15].

Found in the wall of the yolk sac at the end of the third week of gestation, PGCs then migrate through the allantois and the hindgut to the primitive gonads and reside there at 5–6 weeks of gestation. Studies in mice suggest that, during this developmental period, PGCs undergo epigenetic reprogramming including erasure of genomic imprints, DNA methylation and chromatin remodelling, and X-chromosome reactivation in female germ cells, followed later during embryonal development by global re-methylation (for review see [16]).

Although progress has been made, especially during the last decade, the exact mechanism of PGC proliferation in humans is not yet known. Nevertheless, extrapolation from the results of in vivo mouse studies and in vitro differentiation studies has shown that BMP4, leukaemia inhibitory factor (LIF), basic fibroblast growth factor (bFGF), epidermal growth factor (EGF), and platelet-derived growth factor (PDGF) play major roles and can support or inhibit in a species-specific manner important pathways [17,18,19,20,21]. At this point PGCs are still bi-potential and are able to develop into oogonia or gonocytes independent of the karyotypical sex [22]. It is signalling from their surrounding environment that determines sex specification [23].

Once PGCs are enclosed in the developing seminiferous tubules formed by Sertoli cells from weeks 7–9 of development, male sex specification occurs, which is initiated by the expression of *sex-determining region Y* (*SRY*) and *SRY-box* (*SOX*) *9* genes and mediated by fibroblast growth factor 9 (FGF9) and the degradation of retinoic acid (RA) [15,24]. During the first trimester, gonocytes remain mitotically active and still express PGC markers such as POU class 5 homeobox 1 (POU5F1), stage-specific embryonic antigen (SSEA) 4, Nanog homeobox (NANOG), and KIT proto-oncogene receptor tyrosine kinase (KIT). While they slowly lose mitotic activity and PGC markers during the second trimester, they regain some proliferative activity after birth at between 2.5 and 6 months [25].

Since most knowledge addressing human in vivo primordial germ-cell specification and differentiation has been extrapolated from animal studies, in vitro germ-cell differentiation of PSCs offers an opportunity to confirm those results in humans [9]. Although studies mostly performed in mice, rats, and non-human primates have provided major insights into germ-cell development [26,27,28,29,30,31,32], species differences, such as in gene-expression profiles and acting growth factors, as well as the time-spans of specification and differentiation, make one-to-one translations often not feasible.

Following the first report of in vitro germ-cell differentiation from hESCs, seen as spontaneous differentiation into EBs [12], an increasing number of studies are providing us with more detailed insights into human germ-cell development [33]. Nevertheless, in vitro differentiation approaches are highly variable and have been claimed to result in the generation of pre-meiotic up to post-meiotic male germ cells [34].

To investigate the majority of strategies reported for male germ-cell differentiation in this short review, we performed search-engine-based literature studies published between the 1 January 2004 and 31 December 2019 in PubMed and Web of Science in order to summarize the literature regarding in vitro differentiation of human male germ cells from hPSCs, while keeping a special focus on culture conditions, including growth factors and cell lines. For the search-engine-based Web of Science search the following search terms were used: “differentia*” AND “spermato*” OR “germ cell*” OR “primordial germ cell*” OR “gonocyte” OR “germ lineage” AND “pluripotent stem cell*” OR “human embryonic stem cell*” OR “human induced pluripotent stem cell*” OR “human stem cell*”. On the other hand the PubMed search was based on the following terms, including MeSH terms as well as ((((germ cell[MeSH Terms]) OR (primordial germ cells[Title/Abstract] OR germ cell[Title/Abstract] OR spermato*[Title/Abstract]))) AND ((cell differentiation[MeSH Terms]) OR (differentia*[Title/Abstract] OR deriv*[Title/Abstract]))) AND (((pluripotent stem cells[MeSH Terms]) OR stem cells[MeSH Terms]) AND (induced pluripotent stem cells OR embryonic stem cells)). 

Inclusion criteria were the use of hPSCs and differentiation into the bi-potential germ cells state as well as male germ cells, while studies involving the use of animal-derived stem cells and differentiation into oocytes were excluded. Considering only Web of Science, 725 publications could be extracted using the above-mentioned search terms and 1597 publications could be extracted from PubMed. Manual selection after removing duplicates and articles not meeting the eligibility criteria yielded 47 relevant articles which were used to summarize the most relevant protocols for this review.

## 2. Frequently Used Cell Lines in the Course of In Vitro Differentiation Approaches

**Figure 1:**
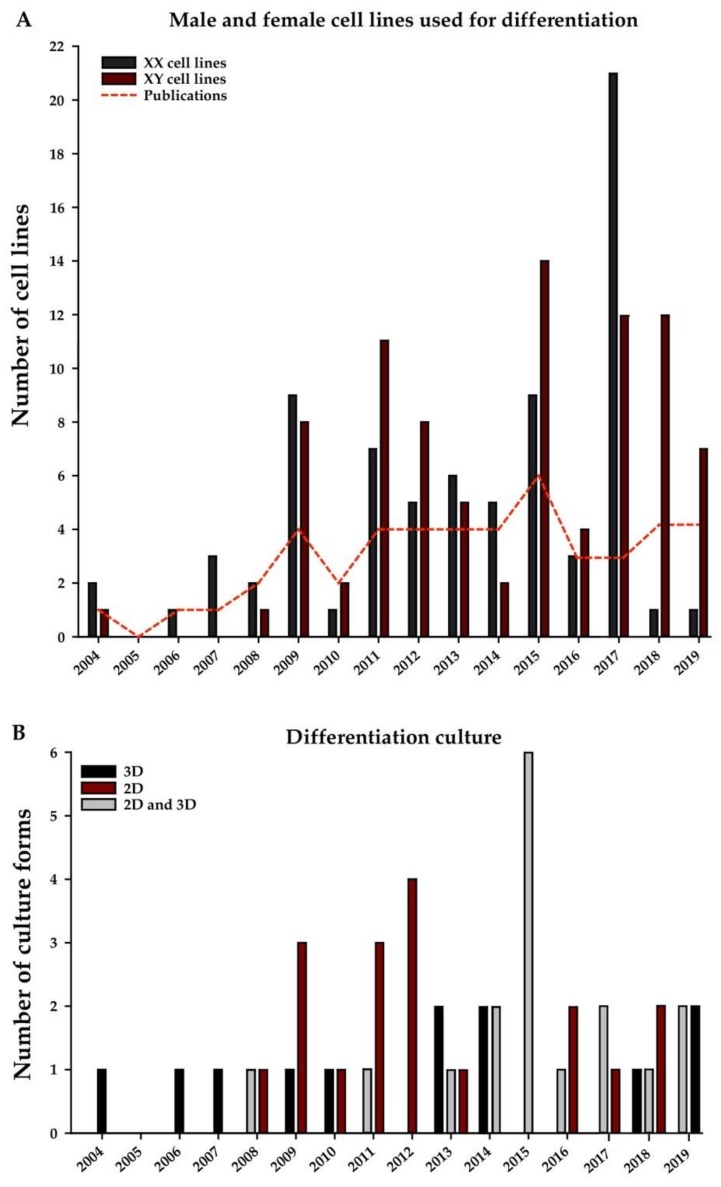
Human pluripotent stem cell (hPSC) lines and culture conditions selected for in vitro human germ-cell differentiation. (**A**) Number of female (black) and male (red) hPSC lines used per year. (**A**). (**B**) Number of different culture approaches reported per year, defined as 3D (black), 2D (dark red), and 3D and 2D (grey) cultures The dotted line shows the number of total publications per year describing the use of these cell lines and culture conditions.

Starting with the first intended in vitro germ-cell differentiation approaches in 2004, both female as well as male PSC lines were used, irrespective of the intended sex differentiation (Figure 1A). Nevertheless, looking at the most recent years, a trend in the use of predominantly male hPSC lines for bi-potential or male germ-cell differentiation can be observed. In vivo, gender-specific differentiation of germ cells is dependent more on the somatic environment, determined by the presence or absence of the *SRY* gene, than the actual germ-cell karyotype. However, humans as well as other mammals, undergoing gonadal sex reversal during early embryo development (e.g., XX males or XY females) suffer from infertility, as their germ cells, although proceeding in maturation to a certain point, are eliminated by check-point mechanisms [22]. Although some differentiation studies show the presence of male-specific markers while using hPSCs with an XX karyotype during in vitro differentiation [35,36,37,38], efficiencies seem to be higher with XY lines. However, further robust progression or functionality of those potentially matured germ cells has not been shown so far.

As differentiation results often vary between different hPSC lines, even with the same differentiation protocol, drawing clear conclusions when comparing the results and efficiency of different protocols is difficult. Although we observed that the hESC lines H1, H7, and H9 were the most frequently used hPSC lines in the original articles covered in this review, most protocols involve the use of in-house-generated hPSC lines, which hampers protocol comparisons. As hESC lines H1 and H9 make up the majority of stem cell line requests from the National Stem Cell Bank, inclusion of commonly available hPSC lines in differentiation protocols could allow better comparison and reproducibility of protocols. Nevertheless, as Merkle and colleagues have shown [39], 5% of 140 tested hPSC lines, among them H9, have acquired somatic mutations. In addition, a recent study by Deuse and colleagues demonstrated the effect of de novo mutations of mitochondrial DNA (mtDNA) during the iPSC generation and culture [40]. Mutations in mtDNA in hPSCs might result in the formation of neoantigens, which will cause an immune response even after autologous transplantation of these cells back into the host. The impact of somatic mutations as well as mitochondrial DNA mutations on human germ-cell derivation is unknown and therefore control lines have to be chosen carefully.

## 3. Variations in Culture Methods

Heterogeneity and comparability of differentiation results is not only due to the use of a great variety of hPSC lines, but also to different culture approaches.

Over the course of time, in vitro germ-cell differentiation approaches have developed from relatively simple one-step protocols towards more complex multi-step approaches (Figure 1B). Starting with EB differentiation in 2004, Clark and colleagues observed that spontaneous differentiation of hESCs allowed the formation of putative germ cells expressing *deleted in azoospermia-like* (*DAZL*) genes, developmental pluripotency-associated protein 3 (DPPA3), DDX4, and synaptonemal complex protein (SYCP) 3, in parallel with other somatic lineages within the EBs ^12^. Since then, EB differentiation protocols, providing a three-dimensional (3D) culture condition, have been commonly applied, either on their own or in multi-step approaches in combination with two-dimensional (2D) culture conditions using defined matrices or feeder cells. Such multi-step approaches were first introduced in 2010 by Richards et al., where hESC-derived EBs cultured on porcine ovarian fibroblasts, among other features, commonly led to the differentiation of DDX4- and BOULE-expressing cells [41]. Thereafter, EBs were further cultured on foetal gonadal stromal cells [42], gelatin [43,44,45], and matrigel [46]. As the most common culture approach, monolayer cultures started being used from 2008 onwards, when Tilgner et al. and West et al. showed that differentiation of hPSCs on gelatin [47] and feeder cells, polyornithine or laminin [48], led to the generation of germ-like cells expressing markers such as SSEA1, DDX4, mutL hHomolog 1 (MLH1), and SYCP3. Since then, monolayer cultures have been further carried out on matrigel [36,37,42,49,50] as well as foetal gonadal stromal cells [42,51]. However, the most complex culture approaches were performed in 2015 by Irie et al. [21] and Sasaki et al. [19], who showed that it might be important to first pre-induce hPSCs to a distinct pluripotent state or a mesoderm-like state instead of jumping to direct germ-cell differentiation from primed PSCs. While Irie and colleagues started their cultures on mouse embryonic fibroblasts (MEFs), with further priming culture on vitronectin/gelatin and final differentiation as EBs, Sasaki and colleagues performed their cultures on laminin 511 with priming on plasma fibronectin and final differentiation as EBs. The gene expression profile of five different human embryonic stem cell lines, which were derived on human foreskin fibroblasts (hFFs), cultured on different matrices (laminin 121 or 521, and Matrigel) was compared, recently [52]. After nine passages on laminin 521, a more homogenous expression pattern of POU5F1, NANOG, SOX2, and growth differentiation factor 3 (GDF3) (all pluripotency markers) could be observed compared to the earlier passages [52].

Although 3D differentiation approaches seem to show better differentiation efficiencies, the manner of culture, might not be as important as the somatic environment in which the differentiation takes place. As PGCs become enclosed by Sertoli cells in the seminiferous tubules or migrate to the basement membrane, being in direct contact with extracellular matrix (ECM) proteins such as laminins and collagens, direct cell-to-cell or cell-to-matrix interactions might be needed for more efficient in vitro differentiation.

## 4. Variation in Growth Factors Supporting Differentiation 

**Figure 2:**
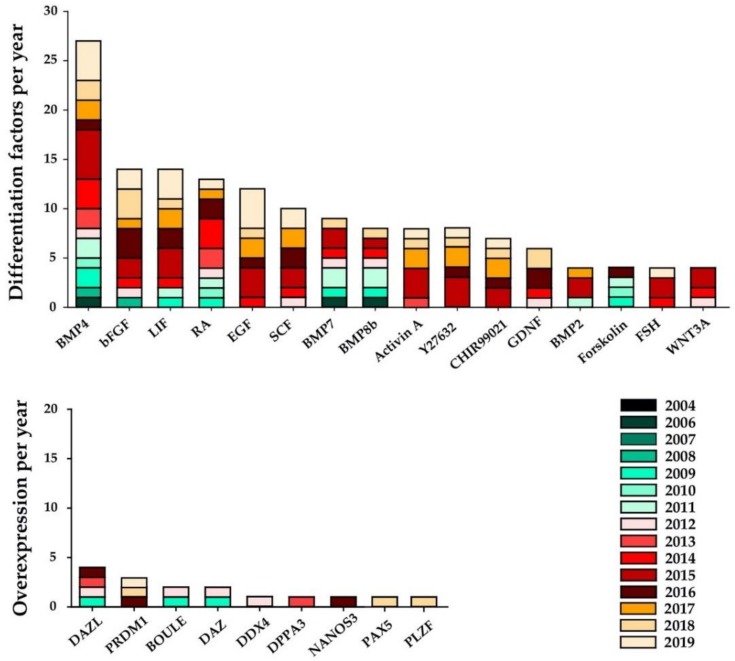
Sixteen most frequently used endogenous differentiation factors for in vitro germ-cell differentiation. Both histograms show the frequency respective endogenous differentiation factors used between 2004 and 2019. Abbreviations: follicle-stimulating hormone (FSH), promyelocytic leukaemia zinc finger (PLZF), paired box protein (PAX) 5.

Although the initial attempts at in vitro germ-cell differentiation were achieved by way of spontaneous differentiation, the variety of factors used to enhance human germ-cell differentiation from hPSCs has increased ever since (Figure 2). BMPs are a group of growth factors that belong to the most commonly used differentiation additives. BMPs and their antagonists have been shown to found specific domains in the developing embryo (for review see [53]). In 2014, BMP4 was found to initiate hESCs to differentiate and self-organize into concentric rings of ectoderm in the middle, extra-embryonic tissue on the outer edge of the rings and endoderm as well as mesoderm in between both layers [54]. It was used in most of the protocols published from 2006 until 2019 [20,35,42,45,47,55,56] to start germ-cell differentiation. BMP2 [19,21,34,57], BMP7, and BMP8b have often been used to replace BMP4 or to achieve an additive effect [19,21,34,36,44,46,49,56,58,59,60,61,62,63,64].

From 2008 on, bFGF started to be used as an additive in differentiation protocols [13,21,38,48,60,62,63,65] while LIF and forskolin were introduced in 2009 [35] and continued to be used in most of the protocols. Retinoic acid, as a meiosis-inducing factor, found its application from 2009 in protocols aiming at germ-cell differentiation beyond PGCs [35,41,60,63,64,66,67,68,69,70,71]. Further, from 2012 onwards, stem-cell factor (SCF), glial cell line-derived neurotrophic factor (GDNF) and Wnt family member 3A (WNT3A) have gained in popularity among differentiation protocols, while small molecules such as SB203580, SP600125, PD0325901, and CHIR99021 as well as Rho kinase (Rock) inhibitor (e.g. Y27632) [19,21] have been used to induce primed hPSCs towards a specific pluripotency stage prior to differentiation [19,21,58,63]. While WNT3A is known to be involved in several developmental processes [72,73] as well as oncogenesis [74], SCF and GDNF are known to be important regulators between somatic cells (e.g., Sertoli cells or peritubular cells) and germ cells [75,76]. However, germ-cell differentiation was not only achieved by addition of endogenous molecules and growth factors but also by over-expression of specific germ-cell-related genes prior to applying differentiation protocols. Examples are over-expression of *deleted in azoospermia* (*DAZ*) [36,49], *DAZL* [36,37,49,63,65], *BOULE* [36,49], *DDX4* [37], *PR/SET domain 1* (*PRDM1*) [63], *DPPA3* [67], and *nanos C2HC-type zinc finger 3* (*NANOS3*) [65]. Nevertheless, in order to improve differentiation protocols, more investigations on the influence of factors produced in the pre-natal testicular stem-cell environment might be needed, as the current knowledge is either insufficient or extrapolated from animal studies. This information would be useful to define effects induced by direct cell contacts with appropriate somatic cells or ECM proteins, in addition to endogenous factors, which might be needed for more efficient or further differentiation.

## 5. Assessment of In Vitro Germ-Cell Differentiation 

**Figure 3:**
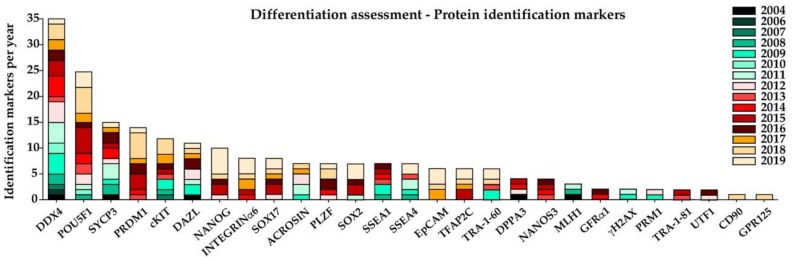
Protein-level germ-cell differentiation assessment. The chart shows the frequency respective protein expression employed in germ-cell differentiation assessment in studies performed between 2004 to 2019, with colour coding for corresponding years. Abbreviation: G protein-coupled receptor (GPR) 125.

Since the first in vitro germ-cell differentiations starting in 2004, the manner of monitoring hPSC differentiation into germ cells has become more and more diverse (Figure 3). While in 2004 evaluation was mainly focused on the bulk gene-expression levels of a few germ-cell-related genes, such as NANOS1, DAZL, DDX4, SCP1, and Tektin 1, in combination with immunohistochemical staining of germ-cell markers, such as DDX4, DPPA3, DAZL, and SYCP3 [12], more than a decade later the assessment of differentiation is becoming more complex. The most recent publications reflect not only multi-step differentiation approaches but also diverse multi-step analysis. Those including fluorescence-activated cell sorting (FACS) for either introduced reporter genes, such as PRDM1, transcription factor AP-2 gamma (TFAP2C) [19], and NANOS3 [21], or surface markers, such as tissue non-specific alkaline phosphatase (TNAP), CD38 [77], EpCAM, and integrinα6 [19]. In order to confirm not only differentiation, but more importantly to establish which genes represent key factors in germ-cell differentiation, bulk or single-cell sequencing should be applied.

Despite the broad range of protein- and gene-expression analyses we observed that DDX4 (expressed in germ cells from 6 weeks post conception), POU5F1 (expressed by PSCs and PGCs as well as gonocytes), and SYCP3 (as meiotic marker) were the three most common protein markers used for the identification of germ-cell differentiation. Further, DAZL (expressed by foetal and neonatal germ cells as well as spermatogonia, spermatids, and spermatozoa) [49], cKIT, PRDM1, SSEA1, and DPPA3 (expressed by PGCs up to gonocytes, as well as differentiated spermatogonia for cKIT) [15,78] and acrosin (the proteinase of the spermatozoan acrosome) [79] were among the top ten protein markers used for identification. Nevertheless, one has to evaluate the results with caution, as, for instance, the haploid cells expressing acrosin, shown in various protocols [13,36,37,49,66], have little meaning without similarities in morphology and periodic acid–Schiff staining, detecting polysaccharides in the spermatid acrosome [80]. Further, we observed that with increased progress in understanding human in vivo germ-cell specification, the spectrum of markers, such as PRDM1, PLZF, SOX17, and NANOS3, is increasing as well, allowing for better distinction of resulting cell types. After Jong and colleagues [81] showed the presence of SOX17 in the absence of SOX2 expression when analysing early human germ cells from foetal testes and ovaries, Irie et al. and Sasaki et al. [19,21] were the first to show the same pattern in in vivo-differentiated primordial-like germ cells.

A gold standard methodology to assess the functionality of germ cells generated from hPSCs as established for animals, including changing DNA contents during meiosis (from diploid to double-diploid to finally haploid DNA contents), as well as the production of zygotes resulting in viable offspring using the in-vitro generated gametes [14], is not available due to obvious ethical restrictions.

## 6. Understanding of In Vitro Spermatogenesis through Single Cell RNA Sequencing

In order to develop and improve in vitro germ-cell differentiation protocols from hPSC towards post-meiotic germ cells, an adequate understanding of the in vivo germ-cell development is crucial. Although the detailed events leading to PGC specification and subsequent maturational steps upon migration into the gonads are still not fully understood in human, recent advances in RNA sequencing (RNA-seq), especially single cell RNA-seq, are gradually increasing the understanding of maturational events as well as the trajectory of specific germ-cell stages from pre-natal to adulthood.

As such, Irie and colleagues were not only able to demonstrate the differentiation of hPSCs towards human primordial germ-cell-like cells (hPGCLCs) but were further able to show the importance of specifically SOX17 in human germ-cell specification by RNA-seq analysis of both, hPGCLCs and human first trimester male germ cells [21]. Similar, Sasaki et al. were able to confirm a similarity of hPGCLCs to human primordial germ cells (hPGCs) as well as monkey PGC by RNA-seq analysis [19].

Nevertheless, RNA-seq is mostly applied in the investigation and understanding of in vivo hPGC states and it has been able to demonstrate a variety of germ-cell stages throughout development in human foetal [82], neonatal [83], infant [84], and adult [83,84,85] germ cells. While early PGC from 4-to 11-week-old embryos show a relative stable transcriptome, adult human germ cells show up to 10 distinct germ-cell populations [85] with up to 4 distinct spermatogonial stem cell (SSC) populations indicating a more complex heterogeneity of SSC populations than A_dark_ and A_pale_ (defined on the basis of the appearance of their nuclei (“pale” and “dark”)), and thereby highlight the challenges related to the differentiation of PSCs into functional male germ cells, present in the post-natal testis.

A further advantage of RNA-seq, in addition to a detailed gene expression profile of different germ-cell types, is investigations of the somatic environment needed to build up the gonadal structure, including the SSC niche as well as the vascular system, and interact with germ cells. Sequencing studies of human foetal [82] and post-natal [82,83,84,85] gonadal cells were able to discriminate between Leydig, Sertoli, and peritubular myoid cells as well as macrophages based on the expression of cell-type-specific marker genes. While 7- to 19-week-old [82] somatic gonadal cells have been shown to exhibit already-distinct cell-type-specific profiles, analysis of neonatal and adult gonadal [83] cells has shown significantly distinct transcription profiles between neonatal and adult Leydig as well as peritubular myoid cells. As most attention on RNA-seq in gonadal tissues is directed towards the germ-cell populations, further investigations focusing on the pre- and post-natal somatic cell types and maturation stages, will be required to understand the somatic cell trajectory as well as the interaction with germ cells during development. Recent studies focussing on in vitro conditions to differentiate SSCs into functional haploid cells, revealed the supportive influence of three-dimensional culture conditions combined with the support of somatic cells present in the post-natal testis [86].

Overall, the recent discriminations of distinct germ-cell populations will potentially allow for improvements of the current differentiation protocols, but most importantly help to determine the similarity of in vitro differentiated germ cells to distinct in vivo germ-cell states.

## 7. Conclusions and Future Perspectives

As male germ-cell differentiation and maturation in vivo is a process spanning many years, starting from epiblast cells specializing and migrating as bi-potential PGCs into the gonad, becoming enclosed in forming seminiferous tubules, migrating towards the basement membrane to become spermatogonial stem cells and starting spermatogenesis after the onset of puberty, in vitro differentiation towards fertile spermatids is one of the most challenging tasks existing in research. 

So far, robust and efficient differentiation approaches are limited, especially when aiming for more advanced stages of male germ-cell differentiation. Further, comparison of various protocols is not only difficult because of the variety of culture approaches but also because of the variety of cell lines used for differentiation, all possessing a specific profile from the start. Nevertheless, great progress has been made during the last decade in the differentiation of hPSCs towards bi-potential PGCs, whereas differentiation towards more advanced stages seems still challenging. Although expression of mature male-specific markers as well as the generation of haploid cells has been shown, morphological similarities to cells at more advanced stages of spermatogenesis, and functionality, are still missing so far.

Future in vitro differentiation approaches employing standardized culture conditions on ECMs, e.g., laminins, will most likely have to include multi-step approaches simulating crucial phases of germ-cell development such as the specification of naïve PSCs towards epiblast cells. Those strategies require robust protocols to derive naïve hPSCs, and transform primed hPSC differentiation towards PGCs, stimulated by BMPs. In addition, effects related to the embedment of PGCs between Sertoli cells such as the suppression of RA during male germ-cell specification need to be addressed as well. A further area of study is the migration of SSCs to the basement membrane with close contact to ECM proteins influencing internal signalling cascades. Finally, the onset of puberty leads to hormonal stimulation of Leydig and Sertoli cells, releasing factors such as testosterone, as well as anti-Müllerian hormone and inhibin B, respectively, allowing differentiation into mature and functional spermatids.

Although in vitro germ-cell differentiation from hPSCs is often mentioned as a potential future option for infertility treatment, one has to bear in mind that the current protocols do not allow sufficient differentiation of fully matured spermatids, and even if possible, testing the ultimate functionality by fertilizing oocytes and producing offspring will present us with many ethical conflicts, which need to be addressed very carefully.

However, strategies aiming in the establishment of models to study the fundamental aspects of germ-cell specification can be considered as crucial steps to gain more knowledge on germ-cell development as well as differentiation. Those are needed to establish strategies for fertility preservation techniques employing hiPSCs from patients without their own germ cells.
